# Perspectives from Alzheimer's Disease Research Centers site directors on the early steps and the lasting impact

**DOI:** 10.1002/alz.71694

**Published:** 2026-09-28

**Authors:** John H. Growdon, John C. Morris, Ronald C. Petersen, Mary Sano, Lon S. Schneider

**Affiliations:** ^1^ Department of Neurology Massachusetts General Hospital Harvard Medical School Boston Massachusetts USA; ^2^ Washington University School of Medicine St Louis Missouri USA; ^3^ Mayo Clinic School of Medicine Rochester Minnesota USA; ^4^ Icahn School of Medicine at Mount Sinai New York New York USA; ^5^ Keck School of Medicine University of Southern California Los Angeles California USA

**Keywords:** Alzheimer's Disease Research Center (ADRC), center program, Clinical Dementia Rating (CDR), clinical trials, mild cognitive impairment (MCI), National Institute on Aging (NIA), NIA research infrastructure

## Abstract

Since the groundbreaking work of Alois Alzheimer in the early 1900s, Alzheimer's disease research has evolved immensely, most notably with the establishment of the Alzheimer's Disease Research Centers (ADRC). Fueled by the vision and funding of the National Institute on Aging (NIA), ADRCs have created a legacy through their work standardizing criteria for Alzheimer's disease diagnosis. Leveraging the experience of the Consortium to Establish a Registry for Alzheimer's Disease, the ADRCs advanced standardized assessment tools, including the Clinical and Neuropsychological Batteries, as well as the CDR, which would make way for uniform data collection, in what we know today as the National Alzheimer's Coordinating Center (NACC). Collaborative data collection was expanded by NIA to support the development of treatments for Alzheimer's disease and related disorders (ADRD) through the Alzheimer's Disease Cooperative Study (ADCS) and the Alzheimer's Clinical Trial Consortium (ACTC) programs. These NIA sponsored programs have created a foundation for today's pharmacological and non‐pharmacological interventions to improve cognition and function in persons living with ADRD.

## Highlights

- The Alzheimer's Disease Research Center (ADRC) program funded by the National Institute on Aging (NIA) has provided decades of support for infrastructure to characterize the trajectory from cognitively normal to dementia in Alzheimer's disease and related disorders (ADRD) with clinical, cognitive, biomarker, and genetic tools.
- Major accomplishments include the advancement of common outcome measures including the Clinical Dementia Rating(CDR) and standardized clinical evaluations that allow collaborative work around the nation and the globe.
- Identification of a pre‐dementia stage, mild cognitive impairment was standardized for use in the Uniform Data Set, allowing researchers across the country and the world to reliably recognize an early stage of disease, a critical step in advancing treatment even before the presence of dementia.
- The center program was the backbone of NIA sponsored clinical trial consortia which advanced instruments, methods and, biomarkers for therapeutic trials for the treatment and management of Alzheimer's disease and related disorders.

## 1 BACKGROUND

The Alzheimer's Disease Research Center (ADRC) program began in the 1980s and was the cornerstone to a focused research emphasis to better understand the etiology and biological contributions to complex dementias of late‐life. The National Institute on Aging (NIA) established five ADRCs in 1984 and another five in 1985, with a goal to create academic centers of excellence devoted to promoting clinical and basic research into the cause(s) of Alzheimer's disease (AD) that would lead to treatments, cures, and even prevention. There was an explicit intention that these centers would catalyze research by attracting regional investigators with relevant scientific and clinical skills to focus on AD. Prior research had been limited and poorly funded; the ADRC initiative was the first concentrated effort to combat this disease. Each of the funded ADRCs had their own expertise in clinical diagnosis, neuropsychological assessments, and neuropathological examinations. It soon became apparent, however, that the ADRCs would be stronger and have a more meaningful impact if they harmonized their procedures together rather than working separately. This section highlights some of these early efforts and documents their legacies.

The Consortium to Establish a Registry for Alzheimer Disease, or CERAD, was one of the key early efforts to harmonize data across the ADRC program.^1^ CERAD specifically was tasked with developing standardized clinical assessments and neuropathological procedures for the diagnosis of AD that would be applied uniformly across the ADRCs. The recommendations were widely accepted and set the stage for the current data repository of the National Alzheimer Coordinating Center (NACC).

Another legacy of the early years of the ADRCs, is the development and implementation of the Clinical Dementia Rating,^2^ a measure of cognition and function that has been standardized and is widely used in research clinical assessments and as an outcome measure in clinical drug trials^3^. Sharpening diagnostic precision was also a mandate of the early ADRCs. Here the concept of mild cognitive impairment was reformulated and continues today as an important diagnostic category in the continuum of cognitive impairment. Treatment was always on the minds of ADRC investigators. A clinical trials group was formed whose mandate was to test new or novel repurposed compounds. The initial study with vitamin E and deprenyl^4^ established the power of collaborative ADRC drug research. Each of these topics is fully elaborated in the sections that follow, addressed from the unique vantage point of founding ADRC Directors who championed these early innovations.

### 1.1 View from founding director: John Growdon

#### 1.1.1 Alzheimer's disease: 1906–1984

AD is recognized today as the most common cause of dementia and one of the most prevalent and serious neurodegenerative diseases^5^; it has widespread recognition in the lay public. Millions of dollars in the United States alone are spent supporting care and research; neurological journals are filled with basic science and clinical reports; and local, national, and international meetings devoted to AD fill the calendar. But it was not always so, as the origin of AD is quite humble. The history, as recounted by Ralf Dahm, begins in 1903 when Emil Kraepelin recruited Alois Alzheimer to join Franz Nissl and others in his department in Munich and challenged them to uncover the biological bases of mental illness and dementia. This quest is an ongoing search that remains unresolved even today. Alzheimer hit partial pay dirt in 1906, when he described neuritic plaques and neurofibrillary tangles in the brain of his 53‐year‐old patient Auguste D. He presented this single case at the 37th Southwestern German Psychiatric meeting where his findings apparently gathered little interest; the November 5 issue of the *Tubinger Chronik* which reported on the meeting devoted only one sentence to Alzheimer's presentation. Several more cases soon followed, and in the 1910 edition of his influential textbook, Kraepelin proposed the name of Alzheimer's disease instead of the initial term pre‐senile dementia; the nomenclature has stuck ever since.^6^

For the next 70 years, there was very little clinical or research interest in AD, possibly because it was widely viewed as a rare form of pre‐senile dementia. Neurology textbooks rarely allocated it more than a few pages and almost nothing was heard at the annual neurological meetings. Publications in journals were rare, although there were a few research voices in this period, the most prominent being Robert Katzman and Robert Terry at the Albert Einstein College of Medicine and Sir Martin Roth and colleagues at the University of Newcastle upon Tyne. Examples include Terry's work on identifying the structure of the neurofibrillary tangle^7^ and the report by Gary Blessed, Bernard Tomlinson, and Martin Roth^8^ linking the number of amyloid plaques in the brain to the extent of cognitive impairment measured during life. Many factors may have played into this lack of interest in AD, but chief among them was that cognitive impairment in older age (“senility”) was common and almost accepted as part of the normal aging process. Specific diseases excepted, the underlying basis for undifferentiated dementia was often described as “hardening of the arteries” and the most commonly prescribed treatments were vasodilator drugs.

In the 1970s, five major events occurred that changed the face of AD research and led to the establishment of the NIA's initiative to establish ADRCs. The first of these was an editorial by Katzman^9^ in which he made two points: there is no difference pathologically between pre‐senile and senile dementia, and the incidence of AD is strongly age‐related, warning of a growing public health crisis as the population ages. These comments shifted thinking about AD as a rare condition to a common one and oriented it toward a medical disease that is potentially treatable rather than an inevitable consequence of aging.

The second major event was authorizing the NIA in 1974, which was under the direction of the first director Robert Butler, to develop a focus on AD. This National Institutes of Health (NIH) institute put AD as a priority on the national health stage and provided federal funds for research, supplementing the relatively small amounts provided by the sister institutes for neurology and mental health. The NIA soon became the major funder of AD‐related projects, although no one at the time could have envisioned the 40‐year history of ADRCs which began with five ADRCs and now number 35 spread across the country. However, even then, the importance was noted by the then Secretary of Health and Human Services, Margaret Heckler, seen in Figure 1 with John Growdon, one of the first Center Directors.

**FIGURE 1:**
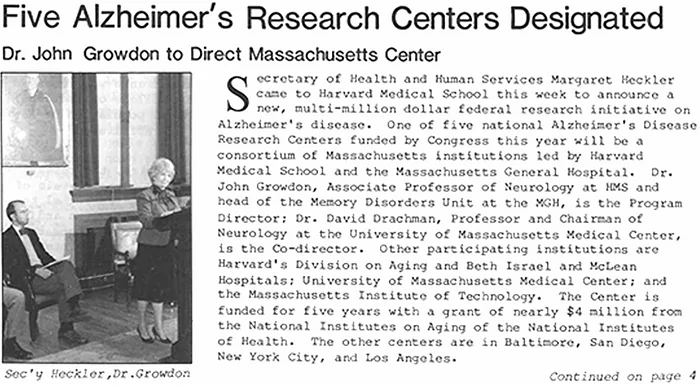
Harvard Gazette 1984 illustration of an article published in the Harvard Gazette which announces the establishment of the ADRC at Harvard, with John Growdon as its director. ADRC, Alzheimer's Disease Research Center.

The third event, complementing the NIA's research mission, was led by Jerome H. Stone, who founded in 1978 the Alzheimer's Disease and Related Disorders Association (now the Alzheimer's Association). This private foundation spread from its base in Chicago to establish chapters across the country dedicated to raising awareness about AD and raising money to support clinical care and research.

The fourth major event was a scientific breakthrough when three separate groups^10^, ^11^, ^12^ independently[Fig alz71694-fig-0002], [Fig alz71694-fig-0003] discovered that indices of acetylcholine metabolism, a neurotransmitter in the brain linked to memory capacities, were reduced in brains of patients with AD. This discovery sparked hope for a new line of treatment and perhaps a cure because drugs can be developed to alter the neurochemical milieu of the brain, whereas the anatomic pathological features of AD seemed immutably fixed. This discovery directly led to the introduction of the acetylcholine esterase inhibitor drugs designed to increase cholinergic neurotransmission that are still a mainstay of treatment today.

The fifth major event changing the face of AD research was a meeting convened under the aegis of the National Institute of Neurological and Communicative Disorders and Stroke (NINCDS) and the Alzheimer's Disease and Related Disorders Association (ADRDA) with the goal of establishing formal criteria for the clinical diagnosis of AD. The resultant report^13^ specified for the first time criteria for definite, probable, and possible AD. These criteria became widely adopted and enabled investigators around the world to share a common understanding of the disease. Having a standard diagnostic language formed a bedrock for the soon‐to‐be awarded ADRCs and ensured that the accomplishments of ADRC investigations rested on a secure foundation. Some of these early accomplishments are detailed in the subsequent sections: further standardized procedures across ADRCs (John Morris); addressing the complexity of dementia diagnosis and promoting the concept of mild cognitive impairment (MCI; Ron Petersen); and the beginnings of therapeutic efforts (Mary Sano and Lon Schneider).

### 1.2 View from a founding clinical core director: John C. Morris

#### 1.2.1 Morris standardizing diagnostic clinical and neuropathological criteria across ADRCs

After joining the newly formed NIA in 1977, Zaven Khachaturian^14^ resolved to develop the infrastructure that would support longitudinal clinical studies of people with AD, which Robert Katzman had recently identified as a largely unappreciated but highly prevalent and malignant age‐associated disorder.^9^ Once he masterfully marshalled the needed NIA resources, Khachaturian established the initial 10 ADRCs in 1984 and 1985 to integrate clinical and basic research regarding AD and to explore the pathogenesis of the disorder.^15^ Each ADRC recruited and followed longitudinally clinically diagnosed participants to generate clinical research data and provide *post mortem* material to confirm the clinical diagnosis. However, each ADRC developed and utilized its own clinical and neuropathological assessment protocols; the diversity of these protocols precluded comparison of results across the ADRCs. Khachaturian and his NIA colleague, Teresa S. Radebaugh, addressed this situation by launching in 1986 the Alzheimer's Disease Patient Registries (ADPR) program, wherein a major aim was to develop standardized diagnostic instruments. Among the funded ADPRs, the multi‐site CERAD (U01 AG06790), was specifically tasked with developing and standardizing reliable and valid assessments of AD to be used by all ADRCs.

The principal investigator for CERAD, Albert Heyman, of Duke University, was an ideal leader. He was well‐known in the AD research field due to his notable investigative contributions but self‐effacingly credited his team for any research success, thus establishing a collaborative and collegial environment. He also was very effective in enabling junior investigators to grow into AD research leaders. Heyman established the Administrative Core, led by himself and his Duke University colleague, Gerda Fillenbaum, to supervise the entire CERAD project, and also formed the Methodology and Data Management Center, led initially by David Mellits from Johns Hopkins University and subsequently by Gerald van Belle from the University of Washington with assistance from his colleague, James Hughes. Heyman then formed the Clinical and Neuropsychology Task Forces, respectively led by John Morris of Washington University and Richard Mohs of the Mount Sinai Medical Center; key contributors to these Task Forces included Christopher Clark of the University of Pennsylvania and Kathleen Welsh‐Bohmer of Duke University. The Clinical Task Force adopted the first published set of clinical diagnostic criteria for AD,^13^ which defined levels of diagnostic confidence as *probable AD*, *possible AD*, and (for the histopathologically verified disorder) *definite AD*. The Task Force's Clinical Assessment Battery aimed to provide experienced clinicians with the minimum information needed to yield a diagnosis of probable AD.^16^ The Clinical Battery incorporated the Clinical Dementia Rating® (CDR®), which determines the presence or absence of dementia and, when present, its severity.^2^, ^17^ The CDR incorporates not only information obtained from the patient but also from the observations of someone who knows the patient well (e.g., the spouse or other family member) to identify whether the individual's cognitive and functional abilities had declined from previously attained levels. The Neuropsychological Battery was administered by psychometricians independently from the Clinical Battery with measures of memory, language, praxis, and general intellectual status. Individual cognitive tests in the Battery included Verbal Fluency (animal naming), a modified Boston Naming Test, the Mini‐Mental State Examination, Word List Memory, Constructional Praxis, Word List Recall, and Word List Recognition.^16^ These tests offered a range of difficulty that facilitated the examination of deficits throughout much of the course of AD.

Standardization procedures were developed so that the Clinical and Neuropsychological Batteries were implemented uniformly in 1987 and 1988 by the original 10 ADRCs and six other dementia centers to assess both individuals with presumptive AD dementia and cognitively unimpaired participants. There was a high level of acceptance of the Batteries by clinicians and neuropsychologists. The Batteries distinguished cognitively unimpaired persons from those with dementia and those with mild versus moderate dementia. Inter‐rater and test–retest reliabilities were substantial. Thus, the CERAD Clinical and Neuropsychological Batteries provided standardized, easily administered, and reliable instruments for evaluating persons with AD.^16^ Subsequently, CERAD established a Neuropathology Task Force, led by Suzanne Mirra at Emory University with valuable assistance from her colleague, Marla Gearing. The Task Force developed a standardized neuropathological assessment of AD based on a semiquantitative rating of neuritic plaque density relative to age.^18^ This protocol established the validity of the Clinical and Neuropsychological Batteries and continues to be used.^1^

CERAD thus developed the first set of standardized clinical, neuropsychological, and neuropathological assessment instruments for AD that have remained in use long after CERAD's funding ceased.^1^ CERAD's uniform assessment protocol allowed for the collection of standardized data to promote collaborative research across the ADRC program. The NIA's Alzheimer's Disease Centers (ADC) program had expanded to 27 ADRCs by the mid‐1990s. To promote comparability among the Centers and further enable collaborative research, a minimum database on a common set of items was developed in July 1997. An Interim Data Coordinating Center (IDCC), under the direction of Denis Evans, at Rush University was established to index the available resources at each ADC, promote AD research, and provide the NIA with information about the ADC program.^19^ The IDCC included elements from the CERAD assessments, including the clinical diagnoses of probable versus possible AD, the Mini‐Mental State Examination, and the CERAD neuropathological diagnostic classification. The IDCC ultimately transferred the database in 1999 to its successor, the Alzheimer's Disease Data Coordinating Center (ADDCC), which was led by Walter Kukull, at the University of Washington. The ADDCC soon was renamed the National Alzheimer's Coordinating Center (NACC), funded by the NIA, aimed to design and implement an expanded dataset to increase collaborative research across the ADC network.^20^ A Clinical Task Force was convened by the NIA to develop with NACC the Uniform Data Set^21^, ^22^ that is obtained with a standardized clinical and cognitive protocol that is administered to all ADC participants at their baseline evaluation and at each annual assessment thereafter.

CERAD successfully fulfilled its mission to develop the initial standardized criteria, definitions, and clinical, neuropsychological, and neuropathologic assessments for AD for use by the ADRC network; its instruments continue to be used.^23^ The establishment of CERAD almost 40 years ago served as the foundation for the current uniform assessment of AD and related dementias that, in turn, has yielded NACC's centralized data repository, the largest standardized Alzheimer dataset in the world. The CERAD instruments, available in approximately 20 translations, are now moving to NACC, where they will continue to be available. The enhanced scientific communication in the early days of NIA‐sponsored AD research that was enabled by CERAD advanced the diagnosis, understanding, and now the biomarkers and treatment of AD.

### 1.3 View from founding director at Mayo Clinic: Ronald Petersen

#### 1.3.1 MCI over the years

The construct of MCI has been around for decades.^24^, ^25^, ^26^ It was originally conceived to describe a stage on the Global Deterioration Scale, Stage 3,^27^ but more recently, the construct was given clinical criteria to assist in its use in clinical practice and research.^28^

The forerunner of the Mayo Clinic Study of Aging (MCSA), the ADPR, led the way for a community‐based study in Olmsted County, Minnesota, and was designed to characterize persons in the community who were experiencing a cognitive decline. Many of these individuals appeared to be unimpaired to the casual observer but had a change in their memory performance that was noticeable to them, but their daily functions were preserved.^29^ These persons had cognitive changes beyond aging but appeared to be normal. This construct was labeled MCI and has been used in the Mayo Clinic ADRC since its inception in 1990, and it has since been adopted by the ADRC program and incorporated into the NACC Uniform Data Set (UDS) criteria.

#### 1.3.2 MCI clinical syndrome

Over the years, international conferences such as the Key Symposia in 2004 and 2012 were held to refine and evaluate the criteria for MCI.^24^, ^30^ Ultimately, the diagnoses were expanded beyond a pure memory impairment to include other cognitive domains yielding single and multiple domain amnestic and non‐amnestic forms of MCI.^24^, ^30^ The NACC program of the ADRCs incorporated the diagnosis of MCI using this framework, and numerous studies have been conducted using the NACC database on MCI.^31^ The UDSv2, UDSv3 and, more recently, UDSv4 have incorporated this construct as a syndrome and coupled it with putative etiologic explanations using biomarkers to yield more precise diagnoses.

In 2004, the Alzheimer's Disease Neuroimaging Initiative (ADNI) was initiated with MCI being the focal diagnostic entity.^32^ ADNI was designed to simulate randomized controlled trials of the time, and since MCI was a relatively new clinical therapeutic target for randomized controlled trials, it was believed to be an appropriate clinical focus.^33^ ADNI took its lead from the Alzheimer's Disease Cooperative Study (ADCS) that had conducted the first MCI clinical trial involving vitamin E and donepezil and followed that design for the implementation of the clinical procedures.^34^

The American Psychiatric Association revised its clinical criteria in the Diagnostic and Statistical Manual – Fifth Revision (DSM‐5) in 2013 and incorporated a new diagnostic condition named “mild neurocognitive disorder” which was essentially MCI.^35^ This was the first venture for DSM criteria to enter into pre‐dementia diagnoses and set the stage for earlier clinical characterization of prodromal cognitive disorders.

#### 1.3.3 AD criteria

Over the lifespan of the ADRCs, there have been several prominent revisions of the criteria of AD. The initial diagnostic framework emanated from the McKhann criteria of 1984.^13^ These criteria were based on clinical features for the diagnosis of AD and served the AD community well for decades. With the advent of AD biomarkers, the NIA–Alzheimer's Association (NIA‐AA) revised criteria for AD in 2011 according to three diagnostic categories: preclinical, MCI, and dementia.^36^ MCI had been one of the central diagnostic syndromes, and it was now being enriched by biomarkers of amyloid and neurodegeneration.^37^ These criteria yielded a probabilistic scheme that the underlying syndromes of preclinical, MCI, and dementia were due to underlying AD. In 2018, another advisory group was formed to revise the criteria in the setting of advancing biomarkers.^38^ This group proposed that AD be defined biologically on the basis of amyloid and tau but acknowledged that the clinical states of cognitively unimpaired (CU), MCI, and dementia represented stages of the progression of the underlying disease. They also introduced a six‐stage scheme of clinical progression for individuals who were amyloid positive with Stages 1 and 2 representing CU, with Stage 2 documenting a change in clinical state while the person was still in the normal range. Stage 3 was essentially MCI, and Stages 4, 5, and 6 represented mild, moderate, and severe dementia, respectively.

More recently in 2024, the criteria were refined to retain the biological features of the definition but allowing for the use of plasma‐based biomarkers for the biological definition.^39^ The clinical stages, including Stage 3 representing MCI, were retained, again, as indices of progression of the disease.

#### 1.3.4 MCI literature

The American Academy of Neurology (AAN) performed an evidence‐based medicine review of the literature on MCI in 2018.^40^ A previous version had been published in 2001 on sparse data,^41^ but over the intervening years, over 11,000 papers had been published on MCI and were reviewed by the AAN committee. The exercise addressed three questions: (1) What is the prevalence of MCI?, (2) What predicts outcome?, and (3) What treatments are available? The study revealed that the worldwide prevalence of MCI in individuals 65 years and older was in the 10%–20% range; the rate of progression to dementia was approximately 10%–15% per year in clinic populations and 8%–10% per year in community populations; and in 2018, the only treatments available were lifestyle modifications such as aerobic exercise.^40^

In the ensuing years, randomized controlled trials have been successful in developing the first United States Food and Drug Administration (FDA) ‐approved disease modifying therapies for AD, aducanumab, lecanemab, and donanemab.^42^, ^43^, ^44^ The clinical indication for all three of these interventions included MCI and mild dementia, and, in fact, MCI was the most common clinical diagnosis for intervention in these trials. Subsequent data have suggested that the earlier these interventions are used in the clinical spectrum, that is, MCI, the more effective the interventions appear to be at slowing clinical progression.^44^ With this rationale, the disease modifying therapies are being evaluated in preclinical (pre‐MCI) persons.^45^

#### 1.3.5 MCI across diverse populations

MCI has been evaluated in under‐represented groups such as African Americans and Hispanics and prevalence figures have varied.^46^, ^47^ In the Health and Retirement Study, the Harmonized Cognitive Assessment Protocol was designed to update figures and documented that MCI was found to be more prevalent in Black and Hispanic individuals compared to non‐Hispanic White participants, raising issues regarding the underlying etiologies.^46^ Some studies have shown that AD biomarkers are actually lower in some under‐represented groups, suggesting other contributing causes to the cognitive impairment found in the prevalence studies.^48^

#### 1.3.6 MCI controversies

MCI has not been without its critics. There are concerns about its imprecision, lack of clarity on the clinical boundaries between normal cognition and MCI and between MCI and dementia.^49^ There is no single “MCI instrument” that the clinician can use to make the diagnosis. The diagnosis ultimately requires clinical judgment. The clinician uses the history from the patient and someone who knows the patient well, performs a clinical examination, perhaps augments it with neuropsychological testing and ultimately comes to a syndromic diagnosis of MCI.

MCI is heterogeneous. Some people with MCI progress to dementia rapidly while others remain stable for considerable periods of time. Yet, others tend to improve. This is to be expected.^50^, ^51^ Many people have inferred that MCI always meant early AD and, as such, all people were expected to progress. However, as noted, MCI is a clinical syndrome and not an etiologic diagnosis. Recent work has emphasized this distinction.^52^ While it is true that many older persons with MCI are likely on the AD continuum and, hence, will progress to dementia at rates of 10%–15% per year, other patterns of progression are to be expected. For example, recently, the entity of limbic‐associated TDP‐43 encephalopathy (LATE) has been described, and in that instance, the underlying etiology is likely TDP‐43, not AD.^53^ Clinical diagnostic criteria for LATE include a prolonged clinical state of an amnestic presentation.^54^ These persons with MCI can look like early‐stage AD but in fact have another underlying pathophysiologic explanation. Their pathology is described as primary age‐related tauopathy (PART), a common brain pathology with neurofibrillary tangles (NFT) that are indistinguishable from those of AD, in the absence of amyloid (Aβ) plaques. In these cases, cognitive impairment is often mild. The collective neuropathology cores of these center programs, required of each ADRC provided the tools to make these exciting neuropathological correlations.^55^

Alternatively, if a treatable cause of the MCI syndrome is discovered, such as untreated obstructive sleep apnea, the person may return to normal function with the treatment of the underlying medical condition.

Finally, an International Work Group (IWG) has proposed the term “prodromal AD” for persons with amnestic MCI who have positive AD biomarkers, and that designation has been useful in a randomized controlled trial environment.^56^ It should be noted that this term refers primarily to amnestic MCI and constitutes a subset of all MCI.

It appears that many clinicians find the concept of MCI and the clinical criteria useful in practice. A survey done by the AAN documented that approximately 84% of its specialists find the term useful, and a corresponding survey in Europe found that approximately 91% of clinicians use the term “MCI.”^57^, ^58^

#### 1.3.7 MCI concluding thoughts

The construct of MCI has evolved over many years in parallel to the maturation of the NIA‐ADRC program. The ADRC NACC database has been extremely useful at supplying the research community with invaluable data on MCI over the course of decades. This has likely contributed to the development of disease modifying therapies for AD. The most recent iteration of the UDSv4 has included the construct of mild behavioral impairment (MBI), which involves early behavioral manifestations of underlying degenerative conditions, and it is complementary to MCI.^59^

MCI has played a role in the advancement of clinical practice and research over the years. It has been a useful steppingstone in clinical progression for persons on neurodegenerative disease spectra. The NIA‐ADRC program has been a test bed and platform on which to evaluate the utility of MCI. As biomarkers for AD have matured over the years, their combination with the clinical syndromic diagnosis of AD has been beneficial for patients on that clinical‐pathophysiologic spectrum.

Early work on subjective cognitive impairment (SCI) as a precursor to MCI was described by Barry Reisberg, Steven Ferris, and Thomas Crook in 1982,^60^ and by Reisberg and Serge Gauthier in 2007.^61^ This condition may be targetable in cases with amyloid pathology. Ongoing work in the ADRCs and elsewhere will determine if this is a treatable condition.

### 1.4 Views from the founding Steering Committee members of NIA sponsored clinical trials consortiums: Mary Sano and Lon Schneider

The establishment of the Alzheimer Center program by the NIA addressed the need to improve detection and diagnosis of AD‐related disorders as described above. The research diagnostic criteria for probable and possible AD were offered by a workgroup sponsored by the NINCDS and the ADRDA,^13^ that provided for relatively accurate recognition of clinical AD before autopsy. At the time, there was a notable paucity of therapeutic development activity. Early agents like Hydergine were widely prescribed, intended for cognitive enhancement in elderly people, yet were minimally studied and provided a limited evidence base.^62^

A clinical trial report of the cholinesterase inhibitor tacrine (THA, Cognex®) was published in 1986 the *New England Journal of Medicine* ^63^ that offered a glimpse of feasibility for treating AD. A substantial reaction to the controversial methods and results of that study led to an NIA and Warner Lambert pharmaceutical company sponsored multicenter trial led by Kenneth Davis, a founding Center Director, and Leon Thal.^64^ The successful progression of this trial demonstrated the feasibility for an ad hoc consortium of academic centers, several from the newly established NIA Alzheimer Centers program, to work together on multicenter clinical trials. This was the nidus for the establishment of the ADCS in 1991. The ADCS was formed as a cooperative agreement between NIA and the University of California San Diego (UCSD). The nascent ADCS engaged most of the NIA ADRCs and ADCs in its efforts, effectively interweaving itself with much of the ADC's program. The ADCS's milestones ranged from conceptualization and advent of multicenter trials in AD for testing novel therapeutics and marketed compounds to the emergence of industry‐shaping innovations and methodologies.

Zaven Khachaturian was a transformative figure in the establishment of the ADCS.^14^, ^15^ He was joined by Neil Buckholtz who later became the director of strategic planning, and together they leveraged visionary leadership and expertise to advance AD clinical trial research. They orchestrated AD‐related activities across the NIH, fostering collaboration and setting strategic priorities. The ADCS was conceived to bridge critical gaps in therapeutic development and to establish robust methodologies for clinical trials.

Khachaturian and Buckholtz were essential in securing funding and building the network of research centers to conduct expansive studies. Recognizing the importance of systematic evaluation, they championed the creation of new diagnostic instruments to enhance trial precision and laid the foundation for collaborative efforts that demonstrated the potential of coordinated research to unravel complex challenges.^65^ Buckholtz became Director, Division of Neuroscience, at NIA and his commitment to open science has left a legacy which endures via the transformative impact of cooperative trials.

Under the leadership of Leon Thal at UCSD, the ADCS operational framework was developed as a multicenter collaboration and multidisciplinary inclusiveness. Through partnerships with academic institutions, usually ADRCs, nonprofit organizations, and the pharmaceutical industry, the ADCS tackled challenges in trial design, such as patient recruitment, endpoint development, data collection and effectiveness. Notably, the ADCS introduced novel methodologies like survival analyses for endpoint determination and actigraphy for remote data collection.

The ADCS expanded their use to MCI and in so doing establishing MCI due to AD as a treatment target.^34^ Beyond pharmacological trials, the ADCS explored non‐pharmacological interventions including behavior management therapy and lifestyle interventions for both agitation and improving function and cognition^66^, antioxidants, anti‐inflammatories, estrogens, and repurposed medications with the goal of modifying the progression of illness.

A major achievement of the ADCS was its contribution to instrument development and standardization for AD trials.^65^ The advancement of tools like the CDR and Alzheimer's Disease Assessment Scale (ADAS) as clinical trial outcomes, and the creation of new scales like the ADCS Activities of Daily Living (ADCS‐ADL) and ADCS Clinical Global Impression of Change (ADCS‐CGIC), advanced outcome measurement in AD trials. Efforts were also made to translate and adapt these tools for broad populations, including Spanish‐speaking communities. Other tools were the Quality‐of‐Life Scale, and the Resource Utilization Instrument designed to capture the economic burden in AD and measure any benefit provided by novel therapeutics. This was followed by a suite of instruments for prevention trials.^67^

The ADCS also conducted the first Alzheimer trial designed to use endpoint and survival analyses,^4^ introduced remote data collection using actigraphy to measure sleep,^68^ and assured that resources were allocated to data and biosamples repositories, which have been shared widely and used to study and improve research methods for subsequent trials.

Following Thal's death in 2006, Paul Aisen continued his legacy of research and innovation. The ADCS diversified its focus with initiatives like the Home‐Based Assessment study,^69^, ^70^ employing emerging technologies for remote evaluations, and secondary prevention trials targeting asymptomatic individuals with amyloid deposits.^71^ More advanced outcomes were developed such as the Preclinical Alzheimer Cognitive Composite (PACC),^72^ a tool bridging clinical and biomarker data analysis.

The ADCS's legacy today is carried forward by two groups. The Alzheimer's Clinical Trial Consortium (ACTC) was launched in 2017 under the sponsorship of the University of Southern California and with a core management team that transferred from UCSD and led by Aisen under a cooperative agreement with NIA. The ACTC integrated lessons from ADCS and other programs to advance experimental therapeutics with imaging, blood biomarkers, and efficient recruitment strategies, and training. It emphasizes large phase 3 and platform trials, investigator‐initiated, NIH funded, and cooperatively with industry. The ACTC also emphasizes education, participant relationships, NIA‐industry partnerships, and sustains momentum toward preventive interventions and disease‐modifying treatments. In fact, these NIA sponsored programs grew a generation of leaders who have worked with industry to advance clinical trial transparency. Examples include the monoclonal antibody trials of solanezumab^73^ and lecanemab^45^ for preclinical AD cooperatively funded by industry and the NIA and carried out by the ADCS and ACTC with lecanemab being the first monoclonal antibody to receive traditional approval by the FDA.^43^

The ADCS continues at UCSD,^74^ under the direction of Howard Feldman, continuing its emphasis on clinical development, phase 2 and 3 trials of new molecular entities targeted for AD and repurposed drugs financed, supported by NIA and industry.

#### 1.4.1 Conclusions on clinical trials consortium

The ACTC and ADCS played pivotal roles in shaping the development of AD therapeutics. Nearly every clinical trial development endeavor has benefited from the tools, samples, and data from these consortia. Thanks to the open science approach, researchers worldwide use their products to advance drug development. The inspired work of NIA has kept the commitment to finding treatments for AD‐related disorders alive, advancing development of therapeutics as summarized in Figure 2. The work has been extended to nearly all centers, summarized in Figure 3.

**FIGURE 2 alz71694-fig-0002:**
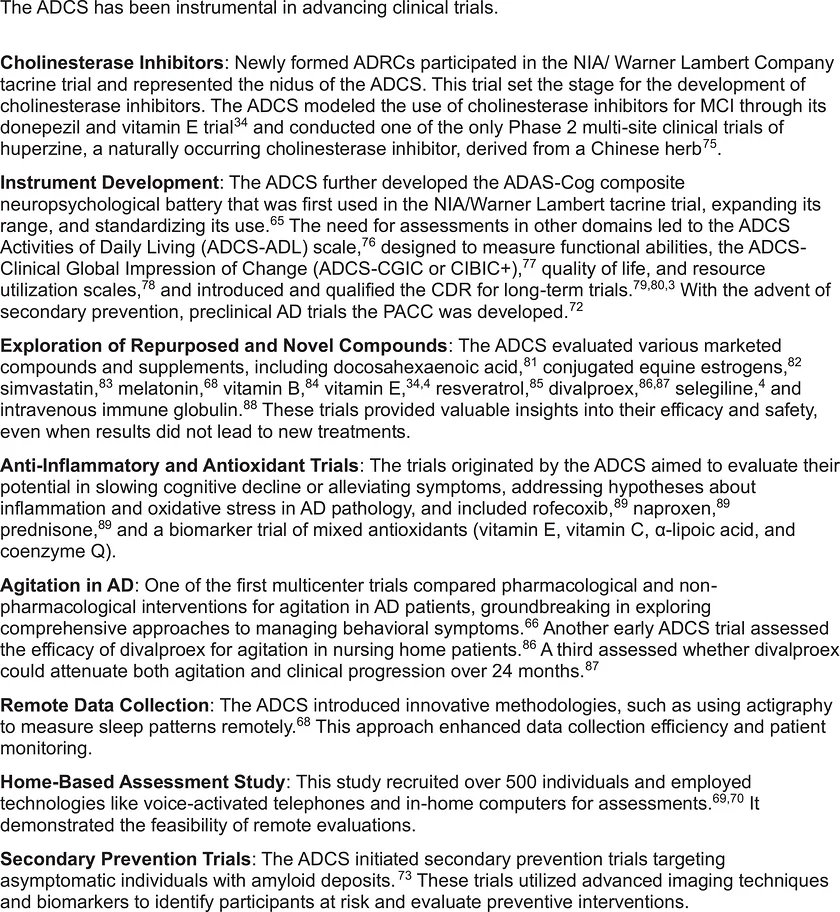
ADCS contributions. A text box listing eight areas where ADCs have been instrumental in advancing clinical trials.^3^, ^7^, ^34^, ^65^, ^66^, ^68^, ^69^, ^70^, ^72^, ^73^, ^75^, ^76^, ^77^, ^78^, ^79^, ^80^, ^81^, ^82^, ^83^, ^84^, ^85^, ^86^, ^87^, ^88^, ^89^, ^90^ ADC, Alzheimer's Disease Center; ADCS, Alzheimer's Disease Cooperative Study.

**FIGURE 3 alz71694-fig-0003:**
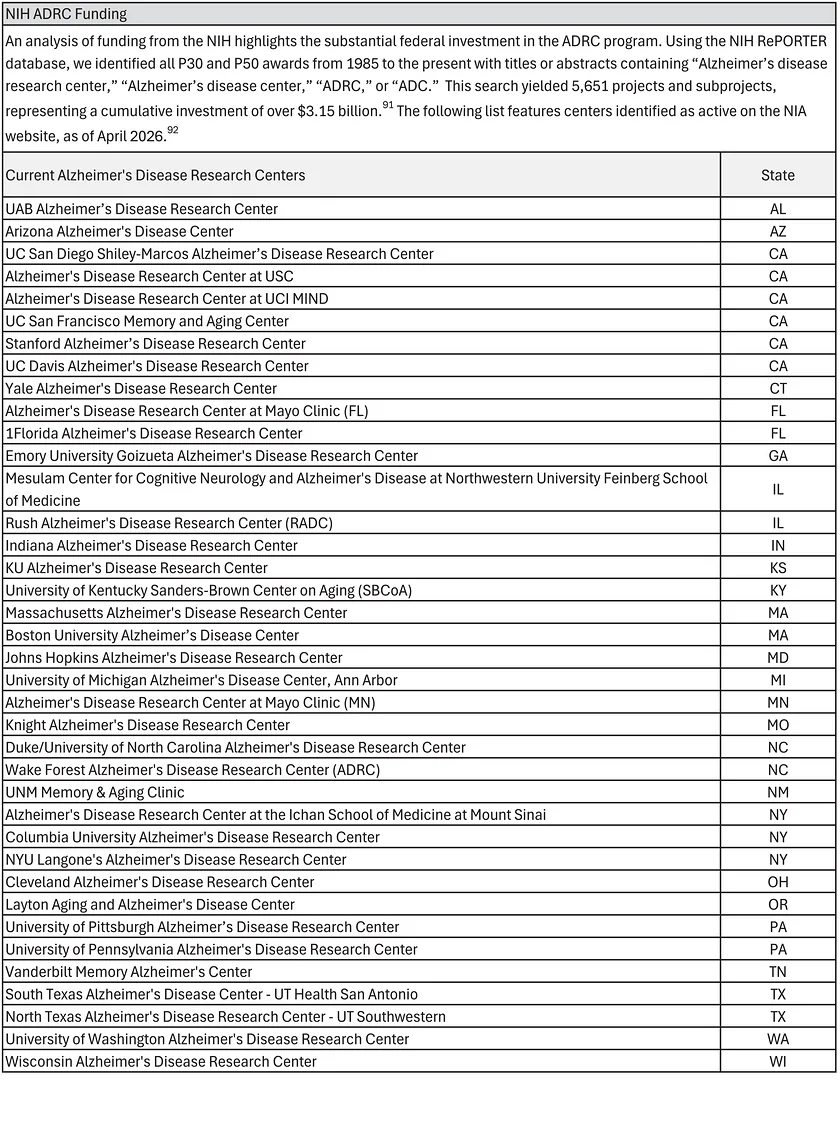
NIH funding and ADRC list. A text box highlighting NIH cumulative funding for the ADRC program and listing all current ADRCs.^91^, ^92^ ADRC, Alzheimer's Disease Research Center; NIH, National Institutes of Health.

#### 1.4.2 Conclusion on NIA accomplishment with center programs and consortia

These collective and very personal perspectives from members of the founding programs to move research forward, hopefully provide an appreciation of the vision of NIA leadership, the commitment of clinician scientists across the country, and the ability to engage public citizens in efforts to address the scourge of ADRD. These efforts, supported by US taxpayers, have led to early detection, better treatments, and informed care. The data collected have supported industry efforts to develop and market better diagnostics and treatments. The programs have trained thousands of researchers and clinicians allowing this legacy of success to continue. This is a story of many heroes and hopefully incredible national pride for what can be accomplished by supporting scientists in the mission of better treatments for so many suffering with ADRD.

## CONFLICT OF INTEREST STATEMENT

The authors have no conflicts of interest related to this manuscript and have disclosed all relationships/activities/interests in the Acknowledgments section. Author disclosures are available in the Supporting Information.

## Supporting information



Supporting Information: alz71694‐sup‐0001‐ICMJE.pdf
